# In Situ Electrochemical Study of the Growth Kinetics of Passive Film on TC11 Alloy in Sulfate Solution at 300 °C/10 MPa

**DOI:** 10.3390/ma13051135

**Published:** 2020-03-04

**Authors:** Lei Zha, Heping Li, Ning Wang

**Affiliations:** 1 College of Chemistry and Materials Engineering, Guiyang University, Guiyang 550005, China; zhalei_gyu@126.com; 2 Key Laboratory of High-temperature and High-pressure Study of the Earth’s Interior, Institute of Geochemistry, Chinese Academy of Sciences, Guiyang 550081, China

**Keywords:** TC11 alloy, passive film, time, high temperature and pressure, EIS, PDM

## Abstract

TC11 alloy is a promising structural material, and has a wide range of applications in many corrosive and high temperature hydrothermal systems. The passive film has an important influence on its electrochemical behavior. In this study, in-situ electrochemical methods (that is, open circuit potential (OCP), linear polarization (LP) and electrochemical impedance spectroscopy (EIS)) were used to monitor the long period electrochemical behavior of TC11 alloy in 0.01 M Na_2_SO_4_ solution at 300 °C/10 MPa. The growth kinetics of the passive film was mainly studied. The correlation between the evolution of the electrochemical behavior and the growth of the oxide film was discussed. The results showed that although the OCP gradually stabilized after twenty thousand seconds, henceforth the polarization resistance (*R*_p_) was still increasing due to the thickening of the passive film. An equivalent circuit was proposed to fit the EIS experimental data, leading to determination of film capacitance and film resistance. Besides, the electrochemical data was interpreted in terms of the point defect model (PDM). The EIS results are consistent with the *R*_p_ results.

## 1. Introduction

As a promising structural material, titanium and titanium alloys are widely used in many fields [1]. Titanium materials can be used in aerospace, marine, automobile industry, chemical industry and biomedical field, because of their excellent corrosion resistance and mechanical properties [2,3,4,5,6,7,8]. One of the reasons for the widespread application of titanium materials is the protective effect of the compact oxide film generated on their surface [9,10]. However, they are still unavoidably subject to corrosion under extreme conditions, such as corrosive high temperature aqueous solutions. The corrosion status of the alloy is closely related to the properties of the surface passive film. Therefore, an investigation about the properties of passive film on the alloys surface can contribute to understand the corrosion behavior of these alloys [11].

TC11 alloy is often used as a structure material for high temperature aggressive aqueous environments, due to its excellent corrosion resistance. TC11 is a suitable material for many hydrometallurgical applications and structural materials for nuclear power plants, as well as for containers for disposal of nuclear fuel wastes. Sulfate is often found in these applications. In addition, a typical temperature of 300 °C is often experienced in many industrial processes, such as nuclear power plant systems, supercritical water oxidation (SCWO) systems, and high pressure acid leaching (HPAL) processes. The purpose of our study is to evaluate the corrosion behavior of the TC11 alloy under high temperature and pressure. 

Our previous researches focused on the electrochemical corrosion behavior of the TC11 alloy at the initial stage of immersion in the presence of sodium sulfate [12,13]. The effects of temperature, pressure and component concentration on the corrosion behavior of this TC11 alloy were investigated [12]. Besides, the electrical properties of the oxide film formed by potentiostatic anodization was also studied [13]. It is also meaningful to monitor the behavior when alloy is immersed in solutions for a long time, because it may have electrochemical characteristics that differ from short-time immersion behavior [14]. 

The film growth kinetics controls the electrochemical behavior variation with time of the facile passive alloy [14]. The aim of our present research is an evaluation of the long-term behavior of TC11 at high temperature and pressure solutions in the presence of a sulfate ion. Its electrochemical response during long time immersion is also dominated by the growth kinetics of the oxide film. There have been many studies on growth kinetics of oxide film on alloy surface [14,15,16,17,18,19,20], and they concluded that the electrochemical characteristics varied over time, especially in the initial stage of immersion. Although the high field model (HFM) is initially adopted widely [21,22], it cannot comprehensively interpret the features of oxide film growth, and it does not involve the problem of stable state thickness [23]. Based upon HFM, a modified model is proposed considering the dissolution [24] and transport behavior occurring at the film/solution interface [25]. Subsequently, the PDM gradually developed and became a common model to deal with the long time growth behavior of oxide film [14,26,27,28,29]. Many researchers used the PDM to evaluate the property variation of passive films formed on iron [30,31], copper [32], stainless steel [33,34,35,36] and nickel alloys [37,38]. However, there are few studies on the properties of passive films formed on titanium alloys. In particular, the long-time electrochemical behavior changes caused by the growth of passive film is a significant issue.

In this present work, the growth kinetics of a native oxide film of TC11 in Na_2_SO_4_ solution in the temperature of 300 °C and pressure of 10 MPa was investigated by a variety of electrochemical techniques, that is, open circuit potential (OCP), linear polarization (LP) and electrochemical impedance spectra (EIS). The effect of the immersion period on the film thickness, film resistance and polarization resistance of the oxide film was studied. The PDM was employed to model EIS data to evaluate the passive film evolution within the immersion time. The related growth mechanisms of the oxide film were also discussed.

## 2. Materials and Methods 

TC11 alloy (Baoti, Baoji, China) was used as the specimen material, and its nominal composition (wt %) is 6.5% Al, 3.5% Mo, 1.5% Zr, 0.3% Si and Bal. Ti. The specimens were fabricated from TC11 rods of a 10 mm diameter. All samples were ground to 1500 grit using SiC papers. The testing solution was 0.01 M Na_2_SO_4_ prepared with analytically pure sodium sulfate (Aladdin, Shanghai, China) and ultrapure water (Institute of Geochemistry, Guiyang, China). High purity argon was used to remove oxygen from the system. 

All electrochemical measurements were performed in a multifunctional autoclave (Institute of Geochemistry, Guiyang, China) equipped with a three electrodes system. The temperature and pressure of the experiments were maintained at 300 °C and 10 MPa, respectively. The counter electrode was a self-made alumina ceramic cone frustum with a platinum wire inside, and platinum paste was sintered on the surface of the ceramic to achieve enough counter electrode surface area. The reference electrode is an external pressure-balanced Ag/AgCl electrode, filled with 0.1 M KCl as an internal reference solution. The reference electrode was placed at room temperature (25 °C) and maintained at system pressure via the Luggin capillary. The thermal diffusion potential problem can be solved based on the work of Macdonald [39]. 

In the present work, the calibrated equation of the electrode potential is shown as follows:

(1) $$\Delta E_{\mathrm{SHE}}\;=\;\Delta E_{\mathrm{obs}}\;+\;0.2866\;-\;0.001\Delta T\;+\;1.745\;\times \;10^{-7}\Delta T^{2}\;-\;3.03\;\times \;10^{-9}\Delta T^{3}$$

where Δ*E*_obs_ is the observed potential of the working electrode vs. the Ag/AgCl external reference electrode, and Δ*E*_SHE_ is the converted potential vs. the standard hydrogen electrode (SHE) at the experimental temperature, Δ*T* = *T* − 298.5 K, *T* is the experimental temperature in K. All the potential values in the text are relative to the SHE except for special mention.

A Princeton applied research (PAR) 2263 electrochemical workstation (AMETEK, San Diego, CA, USA) was used to perform the electrochemical tests. The long-term OCP measurements were performed for 36 h. The potentiodynamic polarization experiment was conducted from −0.3 V vs. OCP to 1.6 V vs. SHE with a scanning rate of 2 mV/s. The EIS were measured using an excitation signal of 10 mV in amplitude in the frequency from 10^−2^ to 10^5^ Hz at OCP. The EIS data were analyzed by a Zsimpwin (3.10) software (AMETEK, San Diego, CA, USA). The polarization resistance measurements were conducted from −20 mV to +20 mV vs. OCP with a scan rate of 0.167 mV/s. All of the electrochemical measurements mentioned above were performed at different immersion time intervals. 

## 3. Results and Discussion

To clear up the evolution of native passive film on TC11 over time, we successively carried out a series of experiments to investigate the variation of the electrochemical status at the electrode surface. EIS combined with LP tests were conducted at certain time intervals during the long-term (36 h) OCP test. Applying a small excitation potential to the electrode will do so in a way that almost does not affect the surface state of the electrode. Hence, we have reason to believe that the mentioned tests will not affect the totality tendency of the long term OCP measurements [14].

### 3.1. Long-Term OCP Tests

**Figure 1:**
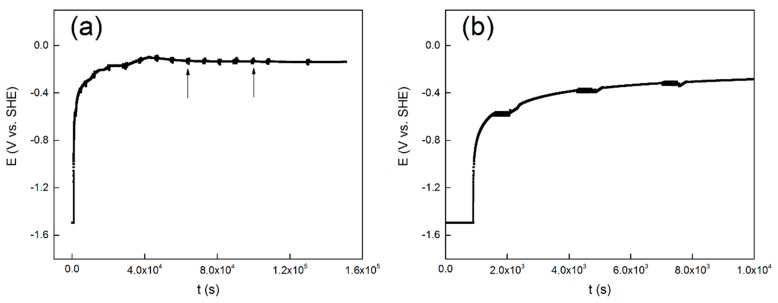
Evolution of open circuit potential with time for the TC11 alloy in 0.01 M Na_2_SO_4_ solution, (**a**) the total 36 h, and (**b**) the first 10 ks.

The evolution of OCP over time is shown in Figure 1a, and the first ten thousand seconds are presented in Figure 1b. Similar evolution of open circuit potential with time was reported for commercial pure titanium [40], Ti-6Al-4V [41] and 304 stainless steel [42]. It shows in Figure 1a that the potential exhibits a rapid shift at the initial stage of the measurement. It also shows in Figure 1a, that at about twenty thousand seconds, the OCP values gradually stabilizes and reaches a steady-state value of −0.15 V. The first ten thousand seconds of the measurement is shown in Figure 1b to get a better view. The black arrows indicate the location of the LP tests and the EIS tests.

It starts with the first cathodic reduction procedure at −1.5 V vs. SHE. Based on the Pourbaix diagram of titanium at 300 °C [43], the metal titanium phase is in a thermodynamic stable state when the pH of the aqueous solution is close to neutral and the potential is −1.5 V vs. SHE. Therefore, a cathodic potentiostatic reduction at −1.5 V vs. SHE for 15 min is believed to partly remove the oxide film formed by air oxidation on the surface of the alloy electrode. Though it is thermodynamically feasible to reduce the oxide film completely, this is unlikely as a result of the slow reduction rate of TiO_2_. Nevertheless, the first cathodic reduction procedure is to reproduce the initial surface condition before the OCP test. Then the OCP values increased rapidly in an exponential form. This is attributed to the formation and development of a passive film on the surface [40]. The protective passive film may act as a barrier layer to prevent the release of the metal ion. After this stage, the passive film begins to thicken over time. At last, the OCP gradually reaches a stable value, and the metal/solution interface reaches a stable state.

**Figure 2:**
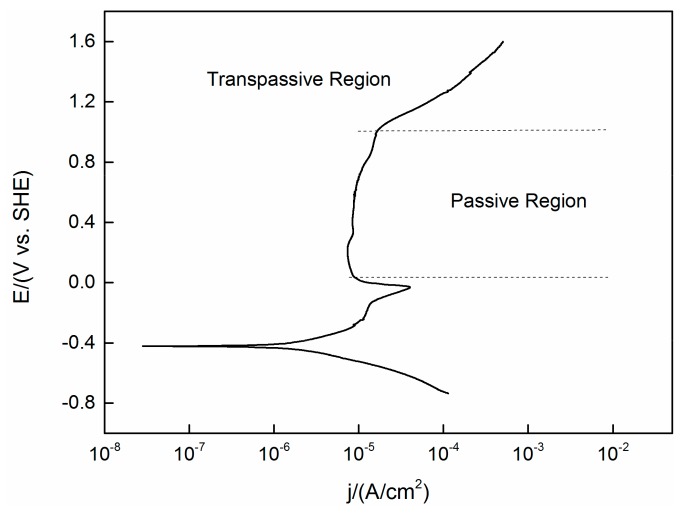
Polarization curve of TC11 alloy in 0.01 M Na_2_SO_4_. Scan in positive direction at 2 mV/s.

A PDP curve obtained under the same experimental conditions (temperature, pressure and concentration) as the OCP test previously mentioned is shown in Figure 2. The steady state OCP values (as shown in Figure 1) are located in the region not yet reach the passive area of the PDP curve. This means that the active–passive transient state has not yet passed, despite the fact that oxides are expected to be present on the surface, it will not lead to complete passivation of the alloy.

### 3.2. Polarization Resistance

**Figure 3:**
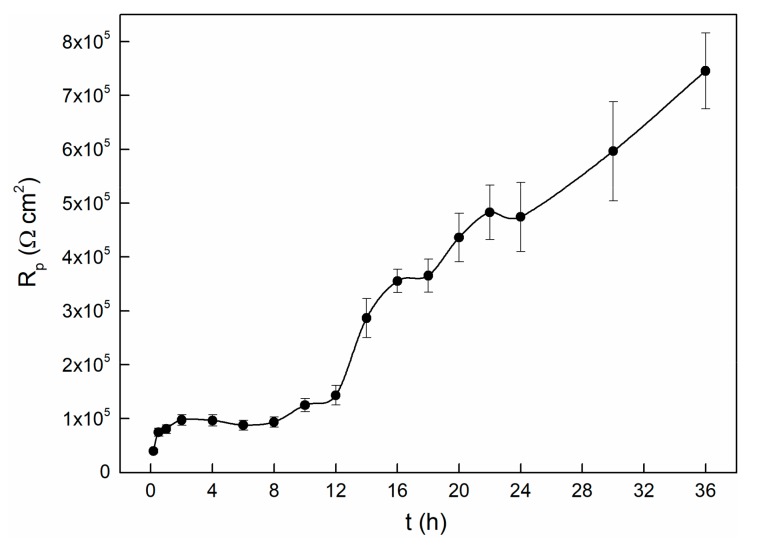
Polarization resistance changes with time for TC11 alloy in 0.01 M Na_2_SO_4_ solution.

In the process of the OCP experiment, LP tests were carried out to characterize the passivation state of the alloy [42]. The evolution of the polarization resistance over time is shown in Figure 3. The varying curve can be divided into three stages. It seems that the *R*_p_ values increase with time in the initial two hours. After that, the *R*_p_ values remain stable for about six hours, followed by increasing very sharply in the remaining tests. This indicates that during this period, the electrode surface is undergoing transition from the activated state to the passivation state, and we thus observed a remarkable increase of polarization resistance [14]. It is interesting to note that although the OCP value is stable after approximately twenty thousand seconds (as shown in Figure 1), the value of *R*_p_ raises obviously after this time node. This indicates that even though the potential reaches a stable state within twenty thousand seconds, the entire interface processes are still in progress, and it changes the passive state. Similarly, the polarization resistance dependence of time of 304 stainless steel also shows this kind of variety. The OCP begins to stabilize at about 15 ks, while the *R*_p_ starts to become stable at about 30 ks. They attributed this to the interface reactions that keep on influencing the structure of the passive film, even though the OCP has reached a steady state [42].

Herein, it is roughly assumed that after twenty thousand seconds, the interface state between the solution and film will keep stable, and therefore the OCP value almost changes no more. Thus, the increases in *R*_p_ value maybe caused by thickening of the passivation film as time prolongs. This speculation can be further verified by EIS because it can obtain more kinetics information and electrode interface structure information.

### 3.3. Electrochemical Impedance Spectroscopy

**Figure 4:**
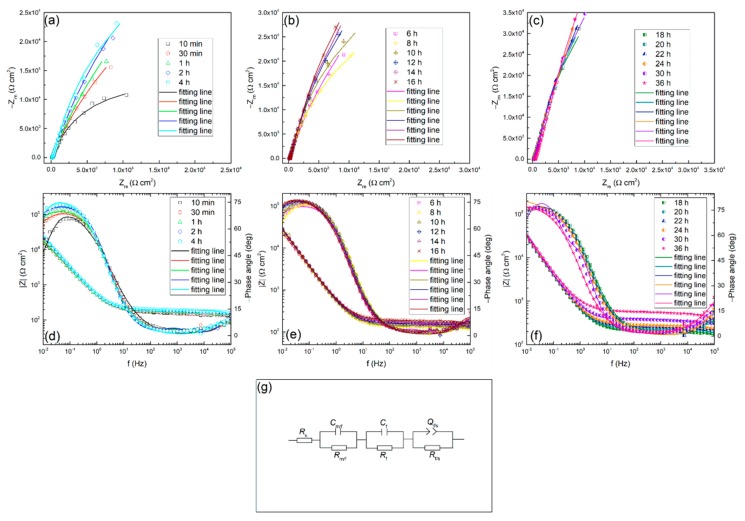
Nyquist (**a**), (**b**), (**c**), Bode (**d**), (**e**), (**f**) plots for TC11 alloy in 0.01 M Na_2_SO_4_ solution as a function of immersion time, and (**g**) electrical equivalent circuit used to fit the electrochemical impedance spectroscopy (EIS) data.

EIS measurements were also conducted and the relevant Nyquist and Bode plots at different times in 0.01 M Na_2_SO_4_ solution are shown in Figure 4. It can be found that all the spectra have a similar shape, which means the same growth mechanism of the passive film at different times during the test. Besides, the semicircular arc of the impedance spectrum changes with time. In the first two hours, the impedance magnitude increases rapidly, then it shows a moderate growth in the next six hours, and then increases continuously with time. The appearance of such a tendency also indicates that the passive state of the alloy surface is changing with time. This is possibly attributed to the growth of oxide film, and it may control the general interface behavior. The above results are coincident with the experimental results of the polarization resistance.

#### 3.3.1. EIS Analysis Using Equivalent Circuit

EIS is a very important method in electrochemical measurement technology. It is an important means to study the kinetics of electrode process, and can provide comprehensive information of interface state and process.

In the current study, the EIS as a function of time of TC11 was continuously measured to understand the growth behavior of the surface passive film. An equivalent circuit is usually used to fit impedance spectrum data to interpret the growth behavior more comprehensively. The EIS data was fitted using a circuit with the circuit description code (CDC) expressed as *R*_s_ (*R*_m/f_ *C*_m/f_) (*R*_f_ *C*_f_) (*R*_f/s_ *Q*_f/s_), which is also presented in Figure 4. Where, *R*, *C* and *Q* represents resistance, capacitance and constant phase element (CPE), respectively. Besides, the subscripts m, f and s refer to metal, passive film and solution, respectively. Priyantha [44] and Nickchi [14,45] used the same circuit in their work. In this work, *R*_s_ means solution resistance, parallel connection combination of *R*_m/f_ and *C*_m/f_ represents the processes occurring between metal and film interface, *R*_f_ and *C*_f_ represents capacitance and resistance of the passive film and (*R*_f/s_ *Q*_f/s_) represents processes taking place between the film and solution interface. The fitting results obtained by using the above circuit are shown in Table 1.

**Figure 5:**
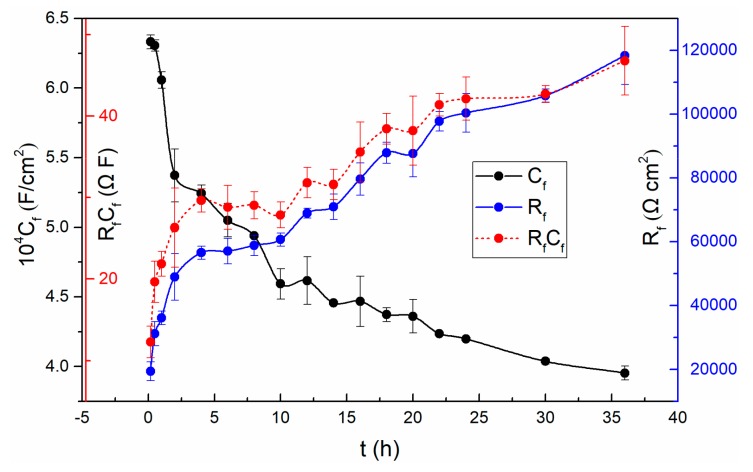
The variation of *R*_f_, *C*_f_ and *R*_f_*C*_f_ with time for TC11 alloy in 0.01 M Na_2_SO_4_ solution.

A lower magnitude fitting error of 10^−4^ indicates that the selected circuit is reasonable [2]. The fitting results show that the solution resistances (*R*_s_) do not change much with time. The *R*_s_ fluctuating around 50 Ω·cm^2^ is due to the solution concentration does not change over time. It is worth noting that the resistance (*R*_m/f_) of the metal/film interface is basically not affected by time until 20 h. So does the corresponding C_m/f_. From 20 h to 36 h, the *R*_m/f_ increases while the corresponding *C*_m/f_ reduces. However, the resistance (*R*_f/s_) at the film/solution interface fluctuates slightly after twenty thousand seconds, which is consistent with the previous discussion in the polarization resistance section. That is, the state of the film/solution interface is stable. The variation of the parameters, *n* and *Y*_0_ of the *Q*_f/s_ is related to the roughness and uniformity of the outer layer of the passive film. Based on the PDM [23], the potential drop across the film/solution interface, *ϕ*_f/s_, is a function of the applied voltage and pH. While the potential drop across the metal/film interface, *ϕ*_m/f_, is a function of applied voltage, pH, the electric field strength of the film and the film thickness. Both the potential drops *ϕ*_f/s_ and *ϕ*_m/f_ are significant, because the potential changes dominate the kinetics of the reactions that occur at the film/solution interface and at the metal/film interface, respectively [46]. In addition, it can be observed that all of the film resistances (*R*_f_) are much larger than other resistances at corresponding times. It indicates that the film evolution dominates the overall interface process. The values of both *R*_f_ and *C*_f_ are shown in Figure 5, and so does the product of them. 

As can be seen from Figure 5, the resistance of the passive film increases significantly at the first stage, and then changes slowly followed by constant increase in the remaining experiments. Since *R*_f_ refers to the resistance of the passive film, the increase of *R*_f_ implies the increasing thickness of the passive film based on the selected circuit. Remarkably, the vary of *R*_p_ value mentioned above is also related to the varying thickness of the passive film. In addition, the decrease of the capacitance (*C*_f_) also proves the increase of the thickness of the film. 

The fact that the *C*_f_ values become smaller while the *R*_f_ values become larger demonstrates that the passive film becomes more and more dense and protective [42].

To find out whether the evolution of *R*_f_ and *C*_f_ are merely associated with the film thickness, the results of *R*_f_ multiplied *C*_f_ are also presented in Figure 5. In this case, Equation (4) can be obtained from the following two general Equations (2) and (3):

*C* = (εε_0_*A*)/*L* (2)

*R* = (*ρL*)/*A* (3)

RC = ρεε_0_ (4)

where, *ε* is the dielectric constant, *ε*_0_ is the permittivity of free space, *A* refers to area, *L* is the film thickness. The product of *R*_f_*C*_f_ will be independent of thickness, and is a constant on condition that the chemical and structural characteristics of passivation films do not change with time [14]. However, the obtained result is not steady over time in the sodium sulfate solution. Thus, the change of *C*_f_ and *R*_f_ is probably not only associated with the film thickness. The above discussions suggest that if the selected equivalent circuit is effective, and accurately represents the physical characteristics of the system, then it is possible that the features of the film will also change with time.

The change in the features of the passive film may be due to the change in the concentration of oxygen vacancy over time [23,47,48,49,50]. The thickness variation of the oxide film involves two reactions: the formation and dissolution of the film [51]. Because the electrode is still in the transition stage from activation to passivation, that is the film is still thickening. This indicates that the kinetics of formation reaction (5) is faster than the dissolution reaction (6) [23].

(5) $$m\;\to {\;M}_{M}\;+\;\left(\frac{\chi }{2}\right){\ddot{V}}_{O}\;+\;\chi e\prime$$

(6) $${\mathrm{MO}}_{\chi /2}\;+\;\chi H^{+}\to \;M^{\delta +}\;+\;\frac{\chi }{2}H_{2}O\;+\;\left(\delta \;-\;\chi \right)e\prime$$

In this case, it may result in a change in oxygen vacancy concentration with time during the test. Consequently, the relative dielectric constant of the passive film will also increase. However, further research is needed to conduct a deep analysis of the EIS data considering the point defects. 

#### 3.3.2. EIS Analysis Using the Point Defect Model

**Figure 6:**
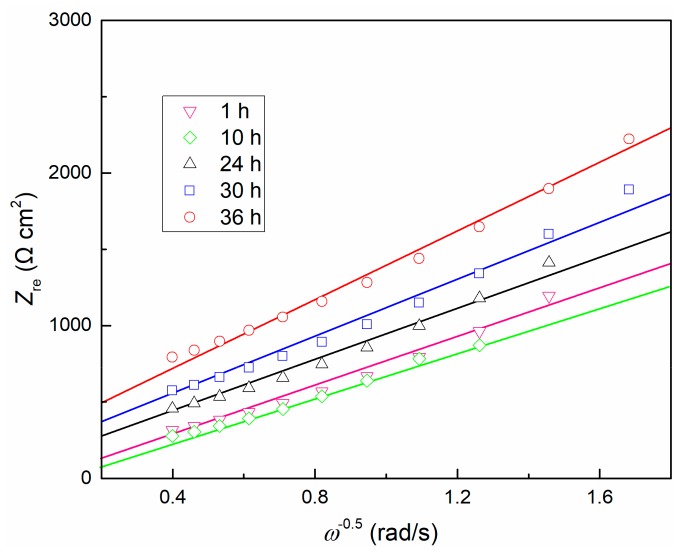
Dependence of *Z*_re_ on *ω*^−1/2^ for TC11 alloy in 0.01 M Na_2_SO_4_ solution.

The EIS data was further analyzed using the point defect model (PDM) [52]. The model predicts that the impedance involves point defects transport in the passive film will exhibit in the form of a Warburg impedance. Because the passive film is electrically conductive, electrons can quickly pass through the film. The impedance of both electrons and electron holes can be expressed as a resistance [52], which leads to negligible contributions of both electrons and electron holes to total conductance, thus the total impedance of the interface is also in a Warburg-type impedance. Correspondingly, the total impedance can be simplified to be proportional to *ω*^−1/2^. Since no redox species is available in the test solution, that is, no redox reaction occurs at the film/solution interface, this situation is undoubtedly reasonable due to the ionic vacancy carrying a much greater amount of current than the electronic current that generates at the film/solution interface [52]. The relationship between real component *Z*_re_ and *ω*^−1/2^ is shown in Figure 6, and it presents a linear dependence. Consequently, compared with ionic conductivity, the electronic conductivity of the passive film becomes insignificant. It is observed that the Warburg coefficients *σ* (equal to d*Z*_re_/d*ω*^−1/2^) increases with time after 1 h. Based on a previous model [28], assuming the TC11 passive film grows inwardly (namely, it is not precipitated from solution), then the transport of oxygen vacancy dominates the growth of the oxide layer, thus the conductance of the cation vacancies, electrons and electron holes can be ignored. Then the values of the Warburg coefficient [52] are equal to:

(7) $$\sigma_{O}\;=\;\frac{RT}{F^{2}\sqrt{32D}\left[C_{{\ddot{V}}_{O}\left(m/f\right)}\right]\left(1-\alpha \right)}$$

where, *D* refers to diffusivity of oxygen vacancy in the passive film, *α* refers to the film/solution interface polarizability, and $C_{{\ddot{V}}_{O}\left(m/f\right)}$ refers to the concentration of oxygen vacancy.

Diffusion coefficient *D* is a function of temperature, and *α* is a fixed value for a certain solution composition [28], resulting in the slope *σ* only related to the change of concentration of oxygen vacancy $C_{{\ddot{V}}_{O}\left(m/f\right)}$. Therefore, it can be inferred that the concentration of oxygen vacancy decreases with time, and thus the internal structure of the passive film becomes more uniform. This is consistent with the prediction of PDM. The product of *R*_f_*C*_f_ mentioned above is also confirmed at this moment. 

At last, we investigated the law for the growth kinetics of the passive film. In general, the generation of an oxide film follows a logarithmic law [28], so does the TC11 alloy. In this study, the data is fitted using the logarithmic growth law deduced from the PDM. It is assumed that the electrical field intensity in the passive film is only related to the electrical and chemical characteristics of the film, and then the assumption of a thickness-independent-field strength is also reasonable. Since the transport of oxygen vacancy causes the growth of the film, in that way, according to PDM, the growth kinetics can be calculated by the following equation:

(8) $$\frac{dL}{dt}=\frac{\Omega }{N_{V}}J_{{\ddot{V}}_{O}}$$

where *L* refers to film thickness, *Ω* refers to the molar volume per cation, *N*_V_ refers to the Avogadro constant and $J_{{\ddot{V}}_{O}}$ is the flux of oxygen vacancy. 

In this case, the thickness of the film can be expressed ultimately in two formulas, depending upon the film thickness, as following [28]:

(9) $$L=\left\{\begin{array}{ll} {\left(\frac{RTA\left(B\;-\;1\right)}{F\varepsilon }t\right)}^{\frac{1}{2}}, & L\;<\;5\;Å\\ \frac{RT}{2F\varepsilon }\left(\ln\left(\frac{2F\varepsilon A\left(B-1\right)}{RT}\right)\;+\;\ln\left(t\right)\right), & L\;>\;5\;Å \end{array}\right.$$

where, *ε* is electrical field intensity. It is worth noting that *A* and *B* is a function of the external voltage and pH. Because the OCP values have reached steady state, the value of *A*(*B*−1) will be a fixed value. For most films, it is reasonable that the thickness is bigger than 5 Å, so the form of logarithmic law was tentatively adopted to reveal the relationship between thickness and time. 

**Figure 7:**
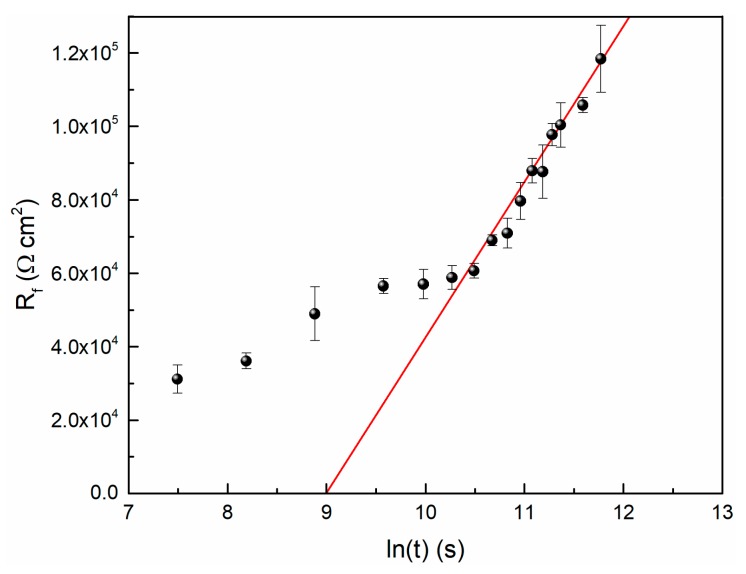
The logarithmic growth behavior of passive film on TC11 Alloy in 0.01 M Na_2_SO_4_.

Based on the simulated EIS data in this study and Equation (3), the relationship between *R*_f_ and time is shown in Figure 7. It is observed that the *R*_f_ data and time could be fitted to logarithmic function, so does the film thickness and time.

If it is assumed that the film resistance is a function of its thickness (that is Equation (3)), then the slope of the fitted line can be used to calculate the value of *ε*. When the value of *ρ* is 10^6^ Ω·m, the result of *ε* is equal to 3.09 × 10^6^ V/cm. Generally, the order of magnitude of the electric field strength of passive film is 10^6^ V/cm [11].

## 4. Conclusions

The long-term electrochemical measurements consisting of OCP, LP and EIS were used to monitor the growth kinetics of passive film on TC11 alloy at 300 °C/10 MPa Na_2_SO_4_ solution. The conclusion is as follows:

The electrochemical response of TC11 alloy changes with time, which is dominated by the growth kinetics of the surface passive film. The OCP increases at initial time, and reaches a stable state within twenty thousand seconds; the reason for this change is related to the formation and growth of passive films. The OCP reaches a stable state within twenty thousand seconds, while the *R*_p_ value still increases significantly after the same time, indicating the continuous growth of the passive film. The polarization resistance data indicate that the corrosion resistance of the TC11 alloy increases with immersion time. EIS data confirm the overall behavior of *R*_p_. EIS analysis using equivalent circuit indicates that the *R*_f_ increases with time while the *C*_f_ decreases as time prolong. EIS analysis using the point defect model shows a logarithmic growth law for the film thickness. Film thickness and oxygen vacancy concentration influence the electrochemical response of passive film over time. The electrochemical data are consistent with the prediction of the PDM.

## Figures and Tables

**Table 1:** The EIS fitting parameters obtained by using the equivalent circuit for TC11 alloy in 0.01 M Na_2_SO_4_ solution.

*t* h	*R*_s_ Ω·cm^2^	*C*_m/f_ × 10^4^ F·cm^−2^	*R*_m/f_ Ω·cm^2^	*C*_f_ × 10^4^ F·cm^−2^	*R*_f_ Ω·cm^2^	*Y*_0,f/s_ × 10^4^ S s^n^·cm^−2^	*n*	*R*_f/s_ Ω cm^2^	*χ*^2^ ×10^4^
0.166	51.05	3.410	350.7	6.332	19,410	4.372	0.6785	2399	1.78
0.5	52.25	4.300	379.0	6.306	31,190	2.095	0.7971	2286	1.49
1	53.56	4.580	384.9	6.058	36,110	2.443	0.6995	2417	1.45
2	46.48	4.759	354.6	5.372	48,990	2.135	0.6973	4037	1.35
4	46.24	4.948	360.5	5.243	56,550	2.073	0.7854	2617	1.18
6	44.06	4.563	337.5	5.050	57,040	2.317	0.7783	3865	1.04
8	41.98	4.452	329.3	4.939	58,830	2.187	0.7454	3845	1.10
10	46.43	4.408	313.3	4.593	60,680	2.346	0.8434	4191	1.16
12	50.55	4.254	359.1	4.617	68,960	1.911	0.7064	3430	1.28
14	56.78	4.021	344.2	4.456	70,940	1.426	0.7716	4280	1.18
16	54.42	3.971	383.7	4.468	79,670	1.522	0.7868	4167	1.18
18	52.47	3.868	387.0	4.373	87,910	1.454	0.8450	4417	1.16
20	58.60	3.889	417.4	4.360	87,650	1.433	0.8270	3013	1.29
22	47.32	3.777	421.7	4.235	97,770	1.301	0.7990	4906	1.14
24	59.16	3.674	448.7	4.199	100,400	1.385	0.8071	3283	2.18
30	55.41	3.415	527.8	4.038	105,800	1.229	0.8180	4217	2.05
36	50.24	3.183	722.3	3.954	118,400	1.247	0.7490	3589	4.84
